# Supplementary material for: Application of Compound-Specific Isotope Analysis in Environmental Forensic and Strategic Management Avenue for Pesticide Residues
Source: Molecules. 2021 Jul 21;26(15):4412. doi: 10.3390/molecules26154412 (PMC8348328; doi:10.3390/molecules26154412)
Supplement: Supplementary file 1 [file molecules-26-04412-s001.zip › molecules-1299794-supplementary.pdf]

Supplementary Information

Review

Application of compound-specific isotope analysis in environmental forensic and strategic management avenue for pesticide residues

Eun-Ji Won [1,2], Hee Young Yun [1,2], Dong-Hun Lee [1,3] and Kyung-Hoon Shin [1,2,*]

[1] Department of Marine Sciences and Convergent Technology, Hanyang University, Ansan 15588, Republic of Korea; ejwon@hanyang.ac.kr (E.-J. W.); heyun2@hanyang.ac.kr (H.Y.Y); shinkh@hanyang.ac.kr (K.-H. S.)
[2] Institute of Marine and Atmospheric Sciences, Hanyang University, Ansan 15588, Republic of Korea
[3] Marine Environment Research Division, National Institute of Fisheries Science, Busan 46083, Republic of Korea; ldh301@korea.kr (D.H.L.)
* Correspondence: shinkh@hanyang.ac.kr; Tel.: +82-31-400-5536

Supplementary Table S1. Recent (2001~2021) review papers on pesticides. (Each study was categorized by main subject, F: fate and/or distribution of pesticide in environments, D: degradation, T: toxicity, M: monitoring, management (remediation) and approaches for it, P: status, suggestion for policy, R: pesticide residues, A: analytical method.)

**Published title**	**Highlights (Subjects)**	**Category**	**References**
Carbofuran toxicity and its microbial degradation in contaminated environments (e.g., carbofuran)	Review on the microbial degradation and its toxicity	F/T	Mishra et al., 2020
Toxicity, monitoring and biodegradation of organophosphate pesticides: A review (e.g., organophosphate pesticides)	Introducing analytical techniques for estimation, and eco-friendly biodegradation approaches	A/D/M/T	Sidhu et al., 2019
Pesticide toxicity: a mechanistic approach	Toxicity and the toxic mechanisms	T	Lushchak et al., 2018
Pesticides, environmental pollution and health	Toxic effects from ecology to human health	T	Özkara et al., 2016
Toxicity of pesticides to aquatic microorganisms: A review	Toxicity of pesticide on microorganisms	T	DeLorenzo et al., 2001
Impact of pesticides use in agriculture: their benefits and hazard	General review on pesticide	P/T	Aktar et al., 2009
Pesticide residue analysis (1999-2000): a review	Residuals effects and characteristics in environments and organisms	A/R	Sherma, 2001
Review of analytical methods for the determination of pesticides in grapes	Analytical methods for residuals in agricultural product	A/R	Grimalt and Dehouck, 2016
A review on occurrence of pesticides in environment and current technologies for their remediation and management	Remediation technologies available for the safer use of pesticides	M/P	Rajmohan et al., 2020
Assessing the safety of pesticides in food: how current regulations protect human health (e.g., glyphosate)	General review for protecting human health and for understanding guideline	P	Reeves et al., 2019
The mobility and degradation of pesticides in soils and the pollution of groundwater resources	Reviews on physical and chemical characteristics of the soil system associated with degradation	D/M	Arias-Estévez et al., 2008
Risk assessment and management of occupational exposure to pesticides in agriculture	Announcing the importance of risk assessment and management of pesticide use for human	M/T/P	Maroni et al., 2006
Pesticides: a review article	Review on adverse side effects of pesticide	T	Al-Saleh et al., 1994

References

Aktar, Md. W.; Sengupta, D.; Chowdhury, A. Impact of pesticides use in agriculture: their benefits and hazards. *Interdiscip. Toxicol.* **2009**, 2, 1-12.

Al-Saleh, I.A. Pesticides: a review article. *J. Environ. Pathol. Toxicol. Oncol.* **1994**, 13, 151-161.

Arias-Estévez, M.; López-Periago, E.; Martínez-Carballo, E.; Simal-Gándara, J.; Mejuto, J.-C.; Garcı´a-Río., L. The mobility and degradation of pesticides in soils and the pollution of groundwater resources. *Agric. Ecosyst. Environ.* **2007**,123(4), 247-260.

DeLorenzo, M.E.; Scott, G.I.; Ross, P.E. Toxicity of pesticides to aquatic microorganisms: A review. *Environ. Toxicol. Chem.* **2001**, 20(1), 84-98.

Grimalt, S.; Dehouck, P. Review of analytical methods for the determination of pesticides in grapes. *J. Chromatogr. A* **2016**, 1433(12), 1-23.

Lushchak, V.I.; Matviishyn, T.M.; Husak, V.V.; Storey, J.M. Pesticide toxicity: a mechanistic approach. *EXCLI Journal* **2018**, 17, 1101-1136

Mishra, S.; Zhang, W.; Lin, Z.; Pang, S.; Huang, Y.; Bhatt, P.; Chen, S. Carbofuran toxicity and its microbial degradation in contaminated environments. *Chemosphere* **2020**, 259, 127419.

Özkara, A.; Akyil, D.; Konuk, M. Pesticides, environmental pollution and health. In: Larramendy, M.L., Soloneski, S. (Eds.), Intech. https://doi.org/10.5772/63094. Environmental Health Risk- Hazardous Factors to Living Species. **2016**

Maroni, M.; Fanetti, A.; Metruccio, F. Risk assessment and management of occupational exposure to pesticides in agriculture. *Med. Lav.* 2006, 97, 430-437.

Rajmohan, K.S.; Chandrasekaran, R.; Varjani, S. A review on occurrence of pesticides in environment and current technologies for their remediation and management. *Indian J. Appl. Microbiol.* **2020**, 60, 125-138.

Reeves, W.R., McGuire, M.K., Stokes, M., Vicini, J.L., Assessing the safety of pesticides in food: how current regulations protect human health. *Adv. Nutr.* **2019**, 10(1), 80-88.

Sherma, J., Pesticide residue analysis (1999-2000): a review. *J. AOAC Int.* **2001**, 84(5), 1303-1312

Sidhu, G.K., Singh, S., Kumar, V., Dhanjal, D.S., Datta, S., Singh, J., Toxicity, monitoring and biodegradation of organophosphate pesticides: A review. *Crit. Rev. Env. Sci. Tec.* **2019**, 49(13), 1135-1187
